# CD47 Blockade Accelerates Blood Clearance and Alleviates Early Brain Injury After Experimental Subarachnoid Hemorrhage

**DOI:** 10.3389/fimmu.2022.823999

**Published:** 2022-02-25

**Authors:** Chao-ran Xu, Jian-ru Li, Shao-wei Jiang, Liang Wan, Xin Zhang, Lei Xia, Xu-ming Hua, Shi-ting Li, Huai-jun Chen, Xiong-jie Fu, Chao-hui Jing

**Affiliations:** ^1^Department of Neurosurgery, The Second Affiliated Hospital of Zhejiang University School of Medicine, Hangzhou, China; ^2^Department of Emergency, XinHua Hospital, Affiliated to Shanghai JiaoTong University School of Medicine, Shanghai, China; ^3^Department of Neurosurgery, XinHua Hospital, Affiliated to Shanghai JiaoTong University School of Medicine, Shanghai, China

**Keywords:** subarachnoid hemorrhage, erythrocytes, microglia, neuroinflammation, mice

## Abstract

**Aims:**

Subarachnoid hemorrhage (SAH) is a devastating stroke subtype. Following SAH, erythrocyte lysis contributes to cell death and brain injuries. Blockage of the anti-phagocytic receptor Cluster of Differentiation 47 (CD47) enhances phagocyte clearance of erythrocytes, though it has not been well-studied post-SAH. The current study aims to determine whether anti-CD47 treatment can enhance blood clearance after experimental SAH.

**Methods:**

The prechiasmatic blood injection model of SAH was used in mice. Mice were either treated with the CD47-blocking antibody or IgG as control. The effect of the anti-CD47 antibody on blood clearance and neurological function following SAH was determined. Neuroinflammation and neuronal injury were compared between the treatment and control samples on day 1 and day 7 after SAH using flow cytometry, immunofluorescence, Fluoro-Jade C, and Nissl staining, RT-PCR, and Western blot analysis.

**Results:**

CD47-blocking antibody sped-up blood clearance after SAH, and resulted in less neuronal injury and neurological deficits than control samples. Microglia played a role in the anti-CD47 blockade. Following SAH Following SAH, CD47 antibody-treated mice had less neuroinflammation and lower levels of apoptosis compared to controls and both one and 7 days.

**Conclusions:**

CD47 antibody treatment has a neuroprotective effect following SAH, by increasing blood clearance rate and reducing brain injury. These findings suggest CD47 antibody treatment may improve SAH patient outcomes.

## Introduction

Subarachnoid hemorrhage (SAH) is a devastating disease associated with high mortality and morbidity in patients worldwide (1). Delayed cerebral vasospasm has been considered the major mechanism of brain injury caused by SAH. Recently, however, randomized double-blind controlled studies demonstrated that a reduction of cerebral vasospasm did not reduce mortality or improve patient outcomes (2–4). Several studies have demonstrated that early brain injury (EBI) plays a decisive role in the prognosis of SAH (5). Friedrich et al. (6) found acute activation of apoptosis and neuronal necrosis after SAH, and, using the rat model, that early management of SAH can significantly reduce mortality and alleviate neurological impairment (6). Current studies suggest that EBI pathogenesis after SAH is multifactorial and highly complex, and includes the inflammatory response, excitotoxicity, oxidative stress, cell autophagy, apoptosis, and necrosis (7). Despite persistent poor patient outcomes, there is no effective therapeutic target for EBI.

During a SAH, a large amount of blood is released into the subarachnoid space, and subarachnoid clots can cause EBI (7). The size of the hemorrhage positively correlates with neurological deficits and poor prognosis (8, 9). During the hemorrhage, erythrocyte lysis releases large quantities of free radicals, which cause oxidative stress that directly disrupts cell signaling, causes protein breakdown, DNA damage, and can eventually lead to cell death (10). Rapid and effective removal of erythrocytes, and preventing them from lysing, could therefore potentially mitigate EBI.

The Cluster of Differentiation 47 (CD47) is an integrin-associated protein and is widely expressed on the surface of erythrocytes as a “don’t eat me” signal. CD47 interacts with Signal-Regulatory Protein alpha (SIRPα) on myeloid cells to prevent phagocytosis (11, 12). Mechanistic studies show that the CD47–SIRPα interaction activates tyrosine phosphatase and the inhibition of myosin-II at the site of the phagocytic synapse (13, 14). The medication Deferoxamine could reduce the expression of CD47 after intracerebral hemorrhage, leading to an acceleration of hematoma removal by promoting erythrophagocytosis (15). Additionally, anti-CD47 treatment has been demonstrated to enhance hematoma clearance and improve prognosis in the experimental intracerebral hemorrhage model (swine and rats model) (16–18). However, the effect of CD47 antibody on erythrocyte clearance after SAH has not been so comprehensively studied. Based on this, we aimed to determine the effect of the CD47-blocking antibody on EBI after SAH.

## Materials And Methods

### Animals

Male C57BL/6J mice (n=150), at 8–10 weeks of age (range 23-25g), were purchased from Shanghai Laboratory Animal Co., Ltd. (SLAC). The mice were raised in a controlled environment (12:12 h light-dark cycle, 25 ± 1°C) and housed with water and food *ad libitum*. All experimental procedures were approved by the ethics committee of Shanghai Jiao Tong University and implemented according to the National Institutes of Health guidelines for the Care and Use of Laboratory Animals.

### Experimental SAH Model

**Figure 1:**
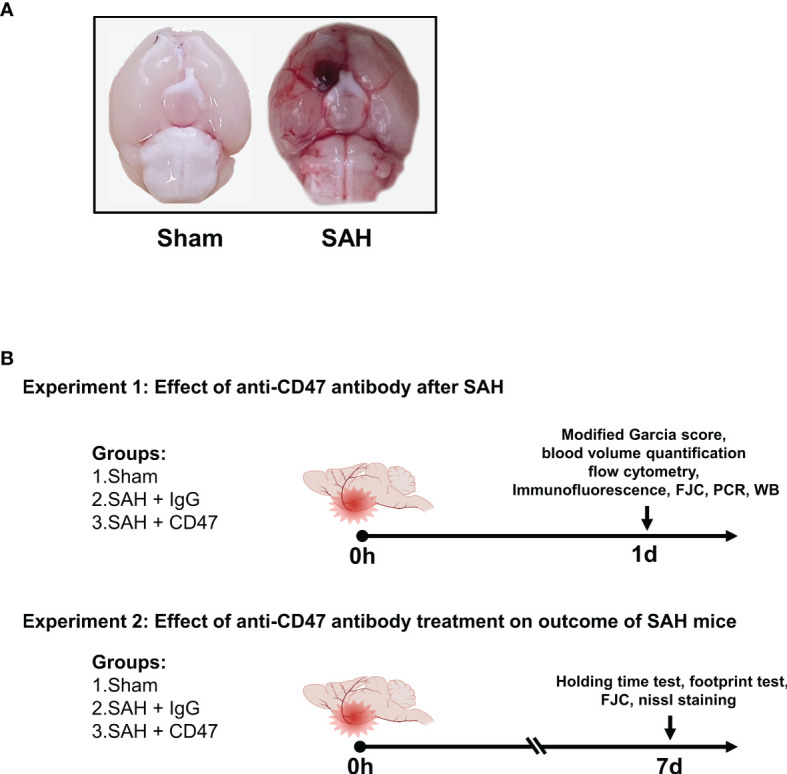
Experimental design. **(A)** Representative brain images of Sham and SAH mice. **(B)** Schematic diagram of experimental design.

The prechiasmatic SAH mice model was created as previously described (19). In brief, with 1% pentobarbital anesthesia, the head of the mouse was fixed on a stereotactic apparatus (Stoelting Co.). The scalp above the anterior skull was then opened with a midline incision and a 0.9 mm diameter burr hole was drilled (4.5 mm anterior from bregma). A 26-gauge needle was then passed through the burr hole to the base of the skull (caudal angel of 40°). A mixture of 10 µg/mL CD47 antibody (Invitrogen, 16-0479-85) or IgG (Invitrogen, 14-4714-85) with 60 μL autologous blood was injected into the prechiasmatic cisternae. The needle was left in place for 5 min before retraction to avoid backflow. Mice underwent the same procedure, without blood and antibody/IgG injections, served as the sham group. A representative picture of the mice brain of sham and SAH models was shown in **Figure 1A**.

### Experimental Design

The experiment design is shown in **Figure 1B**. For the first part of the experiment (1 day), to evaluate the possible early effects of CD47 antibody treatment of SAH, 96 mice were divided randomly into 3 treatment groups (n = 32 per group): sham, SAH + IgG, and SAH + CD47 antibody (**Figure 1**). The mice were executed one day 1 after treatment. We first analyzed the quantitative analysis of hemorrhage clearance in the brain after SAH. Eight mice per group were used for hemoglobin quantification and neurological tests. Six mice per group were sacrificed for flow cytometry analysis. Six mice per group were used for immunofluorescence and Fluoro-Jade C staining, and six for Western blot analysis to measure apoptosis. RT-PCR was performed to quantify mRNA expression of the four pro-inflammatory target genes (CD16, CD32, IL-1β, and TNF-α) (n = 6 per group).

On day seven after SAH (experiment 2), to determine the potential longer-term effect of CD47 antibody treatment, 48 mice were tested randomly among the 3 treatment groups (n = 14): sham, SAH + IgG, and SAH + CD47 antibody. Neurobehavior was assessed at day 7 post-treatment and performed using 8 mice per treatment group. The remaining six mice per group were used for Nissl and Fluoro-Jade C staining to study the neuronal damage.

### Quantitative Estimation of Residual Blood

The cisternal blood volume was estimated by quantifying the hemoglobin content (20, 21). Briefly, the whole brain (with the arachnoid membrane intact) was homogenized in 1000 μl of distilled water. To generate a standard concentration curve, whole blood was added in increments (0, 0.5, 1.0, 2.0, 5.0, and 10.0 μl volume) to 1000 μl of untreated brain sample lysate. The homogenized brain was centrifuged at 12,000 *g* for 30 minutes and, in a 96 well plate, 20 μl of supernatant was added to 80 μl of Drabkin’s reagent (Sigma-Aldrich) and incubated at room temperature (RT) for 15 min. The optical density of the samples and the standard curve was measured at 540 nm and the hemoglobin concentration was calculated.

### Mice Brain Flow Cytometry

**Figure 2:**
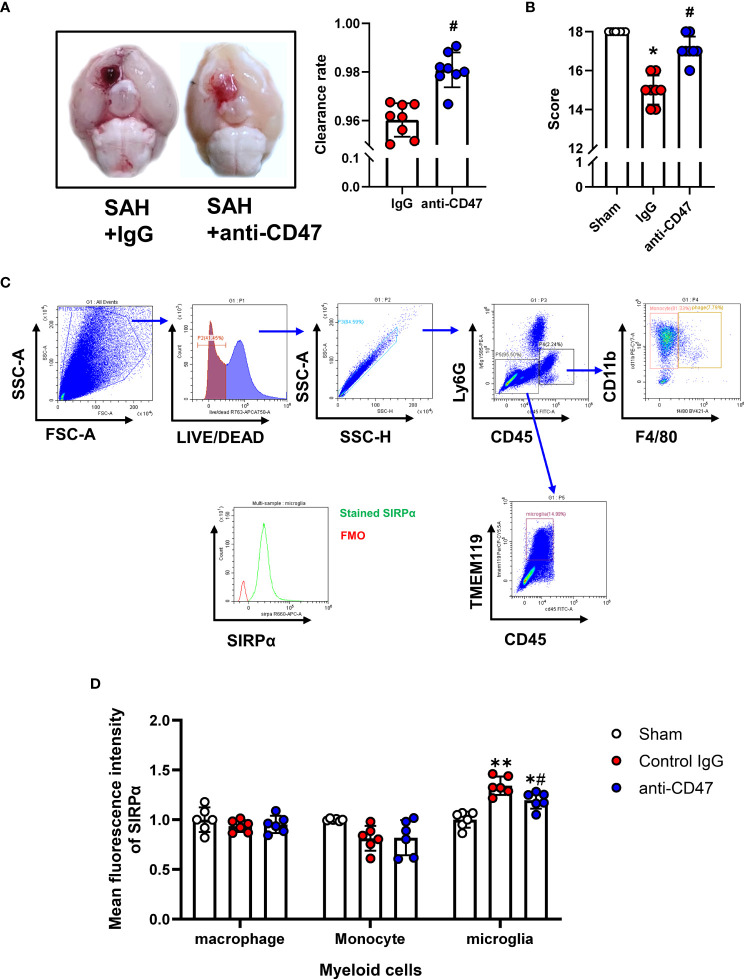
Effects of CD47 blocking antibody on blood clearance and neurological function. **(A)** Representative brain images and quantification analysis of residual hemorrhage in brain samples. n = 8 per group. Mean ± SD. ^#^P < 0.05 *vs.* SAH + IgG group. **(B)** Summary of modified Garcia scores of Sham, SAH + IgG, and SAH + CD47. n = 8 per group. Medians ± IQR. ^*^P < 0.05 *vs.* Sham group, ^#^P < 0.05 *vs.* SAH + IgG group. **(C)** Gating strategy to distinguish monocytes (CD45+, CD11b+, F4/80-), macrophages (Ly6G-, CD45+, CD11b+, F4/80+), and microglia (CD45-, Ly6G-, Tmem119+). SIRPα was then assessed based on FMO control. **(D)** Mean fluorescence index (MFi) of SIRPα in various groups. n = 6 per group. Mean ± SD.*P < 0.05 *vs.* Sham group; **P < 0.01 *vs.* Sham group; ^#^P < 0.05 *vs.* SAH+IgG group.

Flow Cytometry was performed as previously described (22). On the first day after treatment, mice were anesthetized and transcardially perfused with 20 mL of Hanks buffer containing 10 mM HEPES. Brains were then carefully harvested and tissues homogenized with a pre-chilled Dounce homogenizer and filtered through a 70 μm mesh. After centrifugation (340 g for 5 minutes at 4°C), the tissue was resuspended by adding 5ml of 30% percoll (GE Healthcare Bio-science) and then centrifuged at 900 g for 20 minutes. The myelin and supernatant were carefully removed. Red blood cells were lysed using eBioscience™ 1X RBC Lysis Buffer (Thermo Fisher Scientific) before staining. Single-cell suspension staining was performed at 4°C for 30 minutes using the following antibodies: FITC anti-mouse CD45 antibody (BioLegend, 1:100), PerCP-Cy5.5 Tmem119 Monoclonal Antibody (Thermo Fisher Scientific, 1:100), Ly-6G Antibody, PE-Cyanine5 (Thermo Fisher Scientific, 1:100), Brilliant Violet 421 anti-mouse F4/80 Antibody (BioLegend, 1:100), PE/Cyanine7 anti-mouse/human CD11b Antibody (BioLegend, 1:100), CD172a (SIRP alpha) Monoclonal Antibody (Thermo Fisher Scientific, 1:100). Cell viability was assessed using LIVE/DEAD™ Fixable Near-IR Dead Cell Stain Kit (Thermo Fisher Scientific, 1:100). Cells were acquired on a CytoFLEX flow cytometer (Beckman Coulter, United States). Using the flow cytometric gating strategy, shown in **Figure 2C**, we distinguished three groups of myeloid cells, monocytes (CD45+, CD11b+, F4/80-), macrophages (Ly6G-, CD45+, CD11b+, F4/80+), and microglia (CD45-, Ly6G-, Tmem119+). Mean fluorescence intensity (MFI) of myeloid cells was measured using CytExpert software (Beckman Coulter).

### Western Blotting

To investigate the presence of apoptotic signals, tissue of the right temporal lobe of sampled mice brains was homogenized in RIPA lysis buffer (Beyotime) and Pierce Protease and Phosphatase Inhibitor Mini Tablets (Thermo Scientific), and centrifuged (13,000 *g* for 15 minutes at 4°C). Protein concentrations were determined using BCA Protein Assay Kits (Thermo Fisher Scientific). An equal amount of protein samples (40 μg/lane) were loaded onto a 12% SDS-PAGE and transferred onto PVDF membranes (Merck Millipore). The membranes were blocked with 5% milk powder and incubated overnight at 4°C with the primary antibodies: Bcl-2 Rabbit Antibody (1:1000, Cell Signaling Technology #3498) and Bax Rabbit Antibody (1:1000, Cell Signaling Technology #2772) and β-actin Rabbit Antibody (1:10000, Cell Signaling Technology #4970). The PVDF membranes were then incubated with the HRP-linked secondary antibody (1:5000, Santa Cruz Biotechnology) at RT for 1 h. Protein bands were visualized using chemiluminescence reagent kit (Amersham Bioscience). The relative densities of the bands were quantified using ImageJ software (NIH).

### Immunofluorescence Labeling

A series of 8 μm-thick frozen coronal brain tissue slices were taken from brains for immunofluorescent labeling (23). Brain sections were blocked with QuickBlock™ Blocking Buffer (Beyotime) and incubated at 4°C overnight with the following primary antibodies: goat anti–Iba-1 (1:500, ab5076), anti-CD68 (1:150, ab213363) and recombinant anti-Myeloperoxidase antibody (1:1000, ab225474). The tissue sections were washed and incubated with Alexa Fluor fluorescence-conjugated secondary antibodies (1:500, Thermo Fisher) at RT for 2 h. The tissue sections were fixed in DAPI-containing mounting medium and observed using a fluorescent microscope (Olympus). Image superimposition and cell counts were performed using ImageJ software.

### Fluoro-Jade C Staining

Fluoro-Jade C staining (FJC) was used for the specific detection of degenerating neurons. FJC was performed according to the manufacturer’s instruction at 1d and 7d post SAH using an FJC Kit (Biosensis). The FJC positive cells were counted manually using ImageJ software. Three 200 × microscopic fields were counted per sample by investigators blinded to the treatment group. The data were expressed as mean positive cell number/mm^2^.

### Nissl Staining

Nissl staining was conducted to visualize neuronal survival (23). Frozen mouse brain tissue sections were washed and then immersed with 0.5% cresyl violet solution (Sigma-Aldrich). Sections were then sequentially dehydrated in 70%, 80%, 95%, and 100% ethanol, and cleared in xylene for 3 minutes. The brain tissue sections were mounted with Permount and coverslipped. Random fields in the temporal cortex and the hippocampus were observed under a light microscope, and the number of surviving neurons was counted by an investigator blinded to the treatment group.

### qRT-PCR

Total RNA was isolated from brain tissues using TRIzol reagent (Invitrogen) and used for cDNA synthesis using PrimeScript™ RT Master Mix (Takara BioInc). PCR was performed on an Applied Biosystems Quant Studio™ 5 (Thermo Fisher Scientific) and TB Green™ Premix Ex Taq ™ (Takara BioInc). The primers (Sangon Biotech, Shanghai, China) used to measure pro-inflammatory gene expression were listed as follows: CD16 (Forward: ATGCACACTCTGGAAGCCAA, Reverse: AAGAGCACTCTGCCTGTCTG), CD32 (Forward: ATCTGGACTGGAGCCAACAAG, Reverse: TTCTTCATCCAGGGCTTCGG), β-actin (Forward: AGCTCAGTAACAGTCCGCCTA, Reverse: AGGCATTGTGATGGACTCCG),

IL-1β (Forward: GATCCACACTCTCCAGCTGCA, Reverse: CAACCAACAAGTGATATTCTCCATG), TNF-α (Forward: TTGGTGGTTTGCTACGACGTG, Reverse: ATGGCCTCCCTCTCAGTTC).

### Behavioral Examination

A Modified Garcia neurological test scoring system was used to evaluate sensorimotor disorders 24 hours post-treatment (24). Motor functions were evaluated by an investigator blinded to the treatment groups on day 7 to study motor deficiencies using the holding time test and the footprint test (25). Briefly, mice were placed on a 30°-angle-cotton tip wooden applicator, and the duration of the mouse’s stay was recorded. Each mouse was tested three times and the mean duration was calculated. To perform the footprint test, mice were marked with non-toxic paint on their front paws (yellow) and hind paws (blue). A narrow corridor apparatus was used to ensure that the mice walked in a straight line and white paper was used to record paw prints. While walking, if the front and hind paws reached the same level (superimposed), suggesting normal motor function, a score of 0 was recorded. When the left and/or right hind paws could not follow the position of the front paws while walking (no superimposition), suggesting motor dysfunction, a score of -1 was given. Each mouse was measured three times for each paw and the scores were summed.

### Statistical Analyses

The data of modified Garcia score was presented as medians ± IQR (Interquartile range), the rest data were expressed as mean ± SD. Statistical analyses were performed using GraphPad Prism 8.0 (GraphPad Software). Kolmogorov Smirnov test was used for testing normality. Then data were analyzed using a one-way analysis of variance (ANOVA), student t-test, or non-parametric test where applicable. Kruskal Wallis test followed by Dunn multiple comparisons *post hoc* was performed since that data is non-parametric. Results were considered statistically significant at P < 0.05.

## Results

### Mortality

All sham mice survived for the duration of the experiment. In the SAH groups, 11.5% (12/104) of mice died (**Table 1**).

**Table 1:** Mouse mortality among the experimental groups.

Experimental groups	Mortality
*Experiment 1*.	
Sham	0/32
SAH+IgG (1d)	4/36
SAH+CD47 (1d)	3/35
*Experiment 2*.	
Sham	0/14
SAH+IgG (7d)	3/17
SAH+CD47 (7d)	2/16
Total	150
Sham	0/46
SAH	12/104

### Effects of CD47-Blocking Antibody on Blood Clearance and Neurological Function

To study the role of CD47 blocking antibody after SAH in mice, we first analyzed the quantitative analysis of hemorrhage clearance in the brain after SAH. There was significantly less residual blood in the CD47 antibody-treated group as compared to the control group at one-day post-treatment (P< 0.05, **Figure 2A**). The modified Garcia neurological score was significantly lower in the SAH + IgG group compared to the sham group (P < 0.05, **Figure 2B**). However, CD47 blocking antibody administration improved neurological scores (P < 0.05, **Figure 2B**). We then examined the expression levels of the ligand SIRPα of CD47 in different cells by flow cytometry labeling following SAH. Compared with the IgG control group, the SIRPα fluorescence intensity was lower after CD47 antibody treatment, suggesting that CD47 antibody treatment disrupts CD47-SIRPα interaction on microglia, which promoted phagocytic activity (P < 0.05, **Figures 2C, D**).

### Effects of CD47 Antibody on Neuronal Injury 24 h Post-SAH

**Figure 3:**
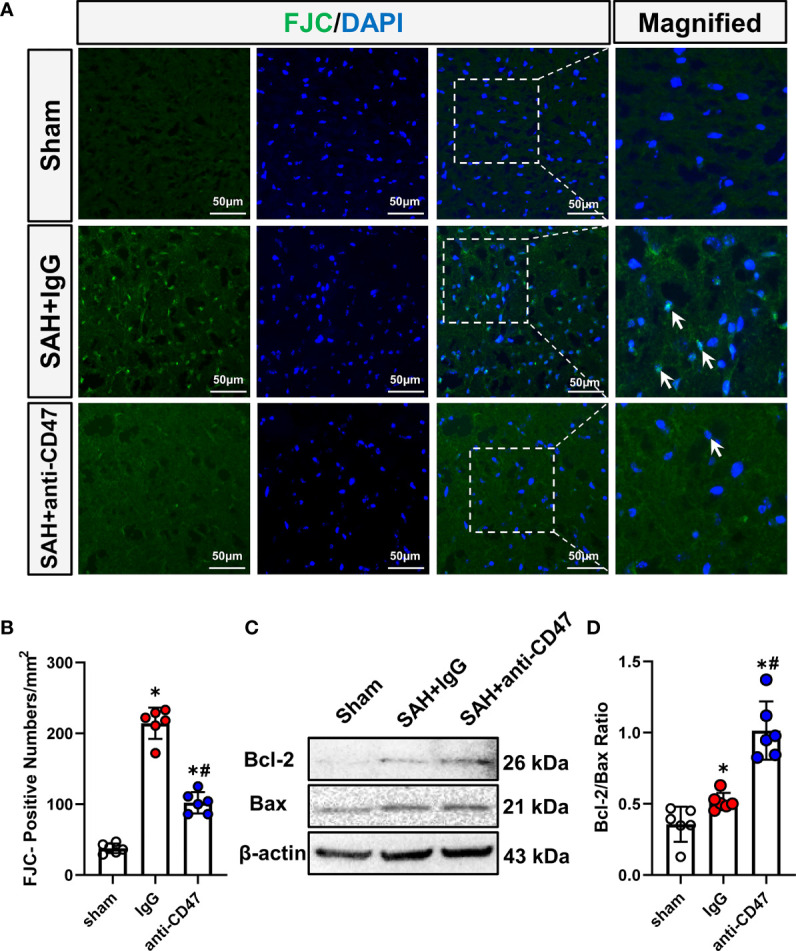
Effects of CD47 antibody on neuronal injury 24 h after SAH. **(A)** Images of Fluoro-Jade C positive cells (indicated by white arrows) in various groups. Scale bar = 50 μm. **(B)** Quantitative analysis of Fluoro-Jade C positive cells per mm2. n = 6 per group. Data are expressed as mean ± SD. *P < 0.05 *vs.* Sham group; ^#^P < 0.05 *vs.* SAH+IgG group. **(C)** Representative Western blotting images shows Bcl-2, Bax, and β-Actin levels in the temporal cortex in various groups. **(D)** Quantification analysis of Bcl-2/Bax ratio in various groups. n = 6 per group. Data are expressed as mean ± SD. *P < 0.05 *vs.* Sham group; ^#^P < 0.05 *vs.* SAH+IgG group.

We next asked whether CD47 blockade could rescue SAH-induced neuronal injury. FJC staining and Western blot for Bcl-2/Bax ratio were conducted to assess neuronal degeneration and apoptosis. After SAH, One day after SAH, significantly more FJC-positive cells were observed. While CD47 antibody treatment group had significantly fewer FJC-positive cells at 1-day post-treatment as compared to the IgG group (P<0.05, **Figures 3A, B**). The Bax has been identified as an indicator of apoptosis, while Bcl-2 is a cellular protein that inhibits apoptosis. Bcl-2 and Bax proteins were detected, and the Bcl-2/Bax ratio was significantly higher in the SAH+CD47 antibody group at 1 d post-treatment as compared to the SAH+IgG group, suggesting that neuronal apoptosis was inhibited in the CD47 group (P<0.05, **Figures 3C, D**).

### CD47 Blockade Suppressed Neuroinflammation and Neutrophil Infiltration

**Figure 4:**
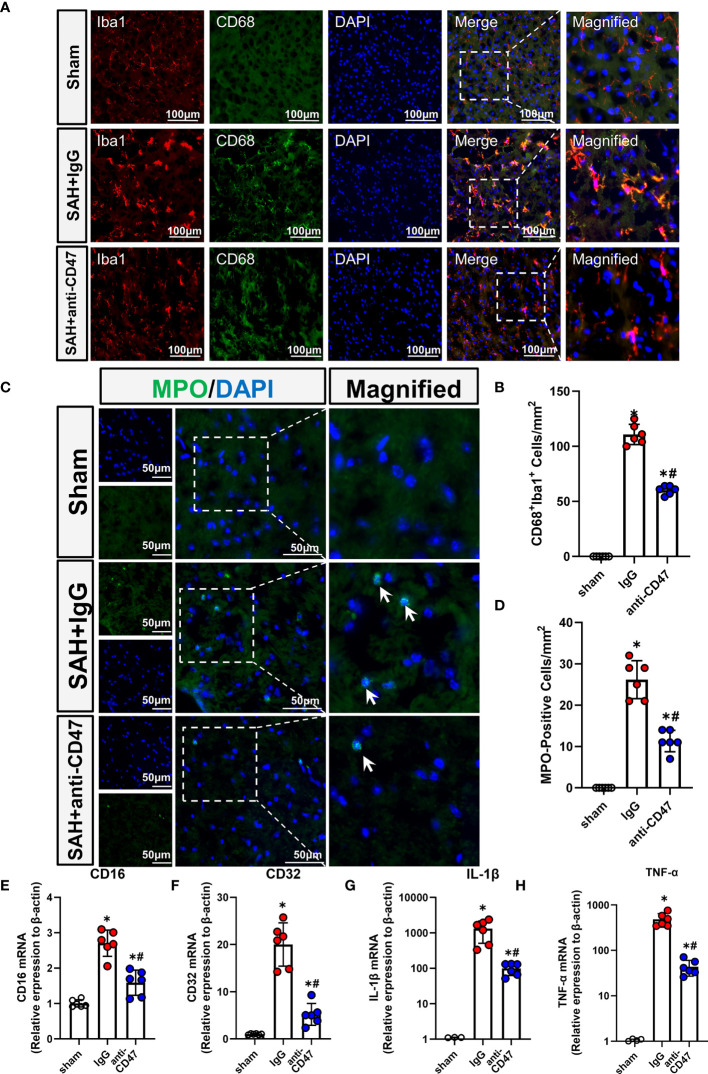
CD47 blockade suppressed neuroinflammation and neutrophil infiltration. **(A)** Images of CD68 immunostaining in various groups, white arrows mark CD68 positive cells. Scale bar = 100 μm. **(B)** Quantitative analysis of CD68-positive cells. n = 6 per group. Data are expressed as mean ± SD. *P < 0.05 *vs.* Sham group; ^#^P < 0.05 *vs.* SAH+IgG group. **(C)** Images of MPO immunostaining (MPO positive cells indicated by white arrows). Scale bar = 50 μm. **(D)** Quantitative analysis of MPO-positive cells in various groups. n = 6 per group. Data are expressed as mean ± SD. *P < 0.05 *vs.* Sham group; ^#^P < 0.05 *vs.* SAH+IgG group. **(E–H)**. The bar graph shows mRNA expression of CD16 **(E)**, CD32 **(F)**, IL-1β **(G)**, and TNF-α **(H)** of brain tissues at 24 h after SAH or sham procedures in mice. n = 6 mice per group. Data are expressed as mean ± SD. *P < 0.05 *vs.* Sham group; ^#^P < 0.05 *vs.* SAH+IgG group.

Activation of microglia/macrophage is considered as a hallmark of neuroinflammation, to study the role of CD47 blockade in neuroinflammation, we identified activated microglia using CD68 staining. There were significantly more CD68-stained microglia observed in the treatment group SAH+IgG, as compared to the sham group, while the number of CD68-positive cells was significantly less in the SAH+CD47 antibody group (P<0.05, **Figures 4A, B**). MPO immunostaining indicated neutrophil infiltration after SAH, and with fewer neutrophils in the CD47 antibody group as compared to the SAH+IgG group. (P<0.05, **Figures 4C, D**). Expression of the pro-inflammatory (M1) associated genes CD16, CD32, IL-1β, and TNF-α, was higher in the SAH+IgG group as compared to control (P<0.05, **Figures 4E–H**). M1-associated genes demonstrated less expression in the SAH+CD47 antibody treatment group as compared to SAH+IgG (P<0.05, **Figures 4E–H**).

### Effects of CD47 Blocking Antibody on Motor Deficiencies Day 7 After SAH

**Figure 5:**
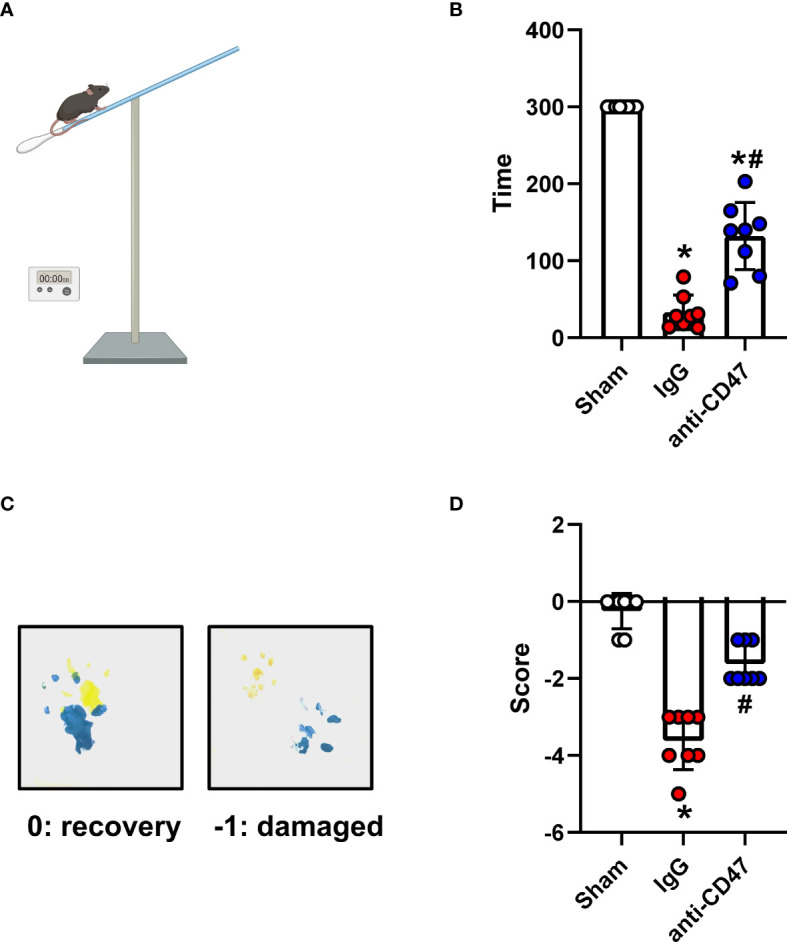
Effects of CD47 blocking antibody on motor deficiencies day 7 after SAH. **(A)** Schematic diagram of the holding time test. **(B)** The holding capacity of holding time test in various groups. n = 8 per group. Data are expressed as mean± SD. *P < 0.05 *vs.* Sham group; ^#^P < 0.05 *vs.* SAH+IgG group. **(C)** Yellow paint on the two forepaws and blue paint on the two hind paws. **(D)** Scores of footprint test in various groups. n = 8 per group. Data are expressed as mean± SD. *P < 0.05 *vs.* Sham group; ^#^P < 0.05 *vs.* SAH+IgG group.

The holding time of mice in the SAH group was significantly less compared to the sham. Mice treated with the CD47 antibody had a significantly longer holding time than IgG-treated animals on day 7 (P<0.05, **Figures 5A, B**). Mice showed a significantly deficient gait after injury compared to the sham group, however, mice in the SAH+CD47 antibody group had relatively less gait deficiency (P<0.05, **Figures 5C, D**).

### CD47 Blockade Attenuated Neuronal Degeneration 7 Days After SAH

**Figure 6:**
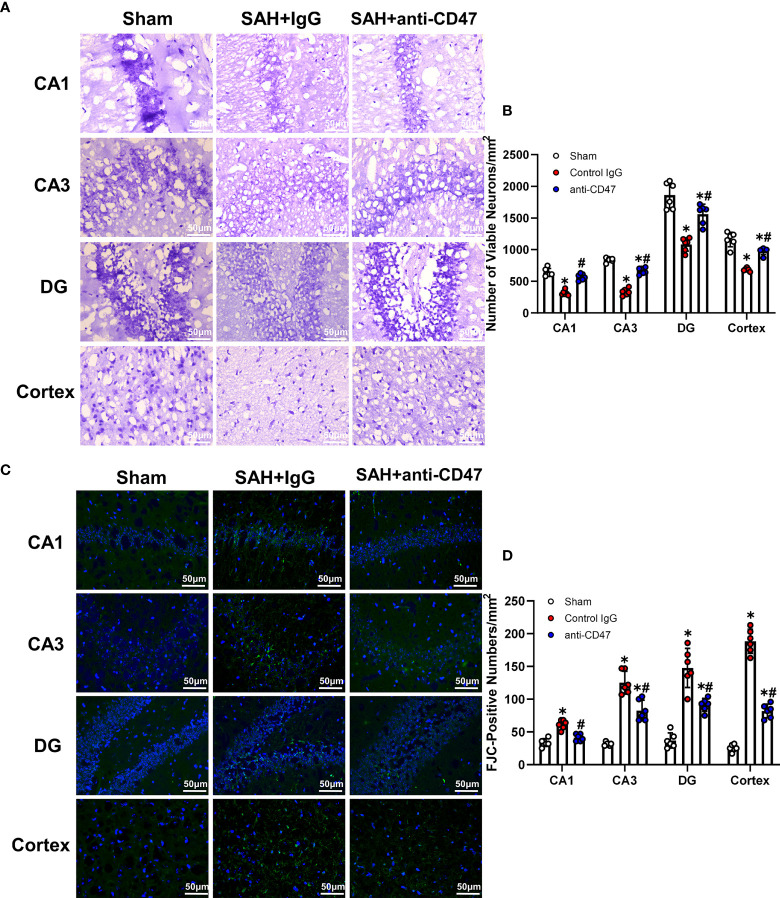
CD47 blockade attenuated neuronal degeneration 7 days after SAH. **(A)** Images of Nissl staining show surviving neurons in the hippocampal regions and temporal cortex sham and SAH groups after 7 days. Scale bar = 50 μm. **(B)** Quantitative analysis of surviving neurons per mm2. n = 6 per group. Data are expressed as mean± SD. *P < 0.05 *vs.* Sham group; ^#^P < 0.05 *vs.* SAH+IgG group. **(C)** Images of Fluoro-Jade C positive cells in the hippocampal regions and temporal cortex in sham and SAH groups after 7 days. Scale bar = 50 μm. **(D)** Quantitative analysis of Fluoro-Jade C positive cells per mm2. n = 6 per group. Data are expressed as mean ± SD. *P < 0.05 *vs.* Sham group; ^#^P < 0.05 *vs.* SAH+IgG group.

Nissl staining and FJC staining was performed to determine whether CD47 could attenuate neuronal degeneration. Mouse brain slices from the sham treatment group demonstrated a significantly lower number of surviving neurons in the CA1, CA3, DG areas, and cortex as compared to the SAH+IgG group, while anti-CD47 treatment reversed this reduction (P<0.05, **Figures 6A, B**). Similarly, FJC-positive cells numbers were more prominent in the hippocampal regions and temporal cortex in both SAH treatment groups as compared to the sham, however, the SAH+CD47 antibody had fewer FJC-positive cells compared to the SAH+IgG group (P<0.05, **Figures 6C, D**).

## Discussion

**Figure 7:**
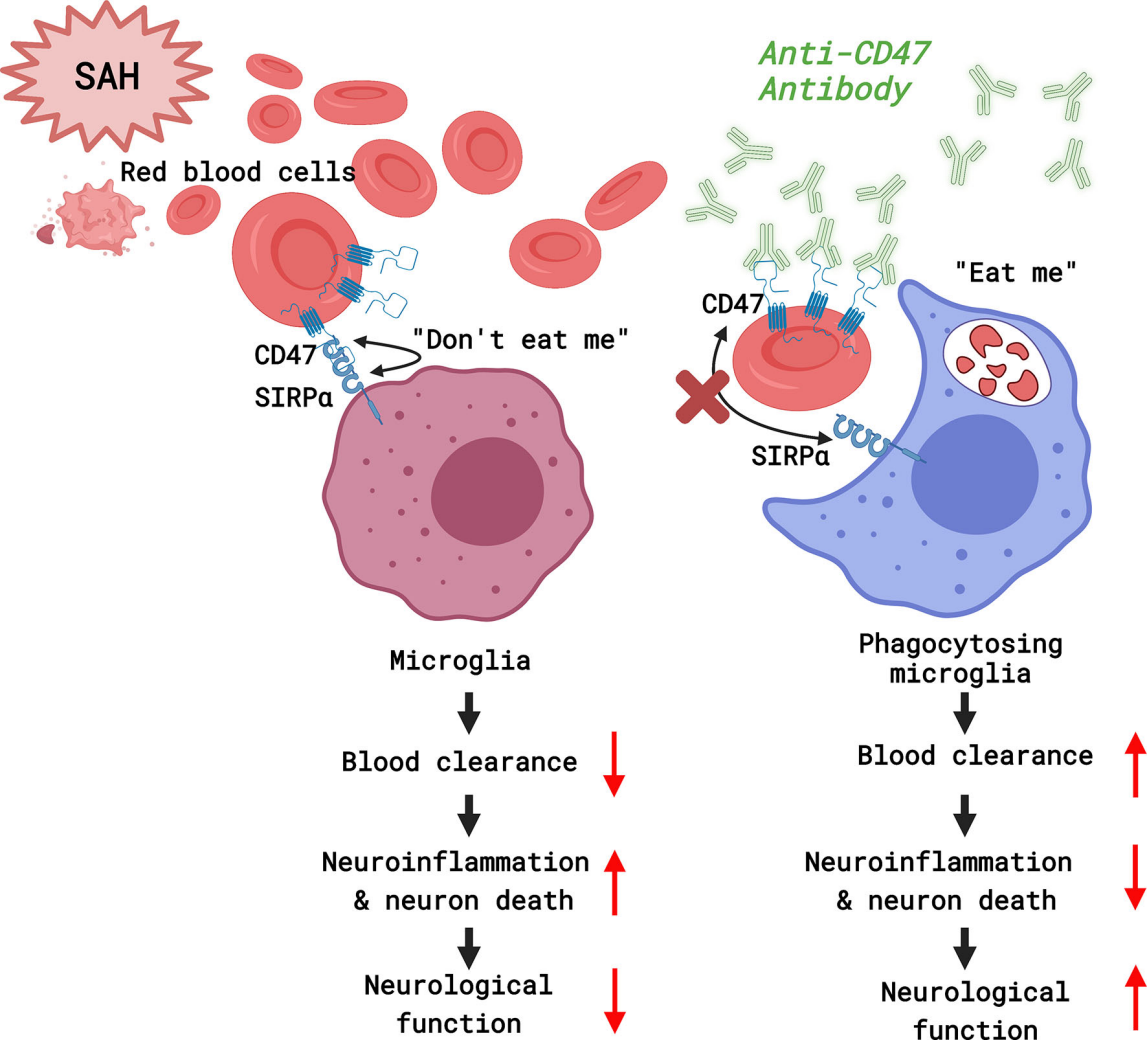
A diagram of the mechanism by which CD47 blockade accelerates blood clearance and alleviates early brain injury after experimental subarachnoid hemorrhage. (Created with BioRender.com).

It was found that CD47-blocking antibodies promoted blood clearance and alleviated SAH-induced neurological deficits and neuronal injury in mice. Microglia are effector cells that respond to the CD47 blockade. Neuroinflammation in response to SAH was attenuated with CD47 antibody, which also facilitated improved neurological function and reduced neurodegeneration by day 7 post-treatment (**Figure 7**).

The autologous blood prechiasmatic injection model of SAH was used because blood distribution is more consistent, better simulating the rupture of anterior circulation aneurysms, than the cisterna magna SAH model (20, 26). The specific quantity of injected blood also makes this model highly reproducible, and the severity of bleeding is less variable than for the endovascular SAH model (20).

The cell surface glycoprotein CD47 (also known as integrin-associated protein) is a marker of “self” that is involved in macrophage signaling regulation (27). As a ligand for the signal-regulatory protein alpha (SIRPα), CD47 can inhibit erythrocyte phagocytosis (11). Our data indicate that blocking CD47‐SIRPα interactions, *via* a CD47 antibody, could enhance blood clearance while conferring a neuroprotective effect in SAH models. SIRPα was expressed on the surface of myeloid cells and indicated that several cells of the myeloid lineage, the brain-resident microglia, infiltrated peripheral monocytes as well as differentiated macrophages, could be the phagocytes in response to SAH. Previous studies suggest that reactive immune cells are mainly from the CNS-resident microglia pool, rather than infiltrating inflammatory monocytes (28) and that murine microglia have the potential to mediate blood clearance after SAH (19). Our findings show that a decrease in SIRPα MFi level of microglia 1d post-SAH, suggesting that the microglia are the main effector myeloid cells after CD47-SIRPα axis disruption. Neuronal injury is considered as the main cause of neurological symptoms in SAH mice (25), CD47 antibody treatment significantly reduced neuronal damage and apoptosis, as compared to SAH+IgG, suggesting a neuroprotective function of CD47 blockage in EBI after SAH.

After SAH, RBC degradation could produce free hemoglobin (Hb), released Hb can be oxidized to oxyHb, methemoglobin (MetHb), and peroxides, which continue to react leading to the formation of Ferryl(Fe4+)Hb, Reactive oxygen species (ROS), and Heme (29). Hemoglobin lysis, therefore, has cytotoxic effects that can lead to neuron death (30). MetHb and FerrylHb, as a result of erythrocyte lysis, are strong pro-oxidants and pro-inflammatory agonists, which initiate inflammatory cell chemotaxis and neuronal necrosis or apoptosis, such as by activating NF-κB (31). Heme can also trigger the neuroinflammatory pathways, through the TLR4 signaling pathway, by activating microglia to produce TNF, IL-6, and IL-1β, leading to neurological damage (32, 33). We found that CD47 antibody treatment promoted blood clearance, then we hypothesized that reduction of erythrocytes in the subarachnoid space may help to suppress the neuroinflammatory and signaling cascade thereby alleviating EBI after SAH. Microglia/macrophage activation, as well as neutrophil infiltration, are central to the inflammatory response following SAH (25). Microglia/macrophages of the central nervous system can polarize into two states in response to injury, termed the classical phenotype (M1-like phenotype) and the alternative phenotype (M2-like phenotype) (34). Van Dijk et al. (35) found that microglia can exhibit phagocytic activity in early brain injury after SAH, thereby reducing the expression levels of IL-1, IL-6, and TNF-α, and ultimately reducing neuronal apoptosis (35). In this study, CD47 antibody treatment reduced the M1 polarization marker (CD16/32, IL-1, TNF-α), decreased neutrophil infiltration and microglia/macrophage activation, suggesting CD47 involvement in orchestrating SAH-induced neuroinflammation, which confirms our previous hypothesis.

Neuroinflammation during EBI is related to the increase in neuronal death, often reflected by the development of neurologic deficits and referred to as Delayed cerebral infarction (DCI), typically occurring on day 4 to 7 after SAH (36). Erythrocyte lysis is involved in the majority of DCI pathological processes following SAH (29). Palade et al. (37) suggested that neuronal apoptosis occurs in the cortex, but hippocampal neuronal death may be associated with ischemia (37). We observed motor dysfunction and hippocampal and cortical neuronal damage 7 days after SAH, suggesting that DCI may have occurred which result in neuronal apoptosis and further motor deficits. This result is consistent with previous findings of SAH-induced motor deficits associated with neuronal death in clinical patients and animal models (25, 38, 39). Our results suggest that the CD47 blocking antibody facilitates delayed neuronal damage and motor deficits by promoting blood clearance.

Limitations of this study should not be ignored. First, the impact of the blockade antibody on the sham group had not been determined, which would be more convincing to add the sham + CD47 group as well for comparison. Besides, we focus on the CD47-SIRP axis of microglia in regulating erythrophagocytosis, though other CNS macrophages likely play a role in neuroinflammation. Previous studies have suggested that resident microglia rather than infiltrating macrophages are involved in neuroinflammation and blood clearance after SAH, but recent studies have shown that the immune cells of choroid plexus and meningeal/perivascular macrophages also play an important role in outcome after SAH (40, 41). The exact mechanism of CNS border-associated macrophages (BAMs) remains to be elucidated. Also, the effect of sex on CD47 blocking antibody treatment was not explored and could be investigated in future studies.

In conclusion, our data revealed that the CD47-blocking antibody can enhance blood clearance and reduce brain injury in the SAH mice model. These results may help to provide strategies for the prevention, or treatment, of EBI after SAH. As such, it could lay the foundation for the application of CD47 antibody in translational medicine, leading to better patient outcomes in the future.

## Data Availability Statement

The raw data supporting the conclusions of this article will be made available by the authors, without undue reservation.

## Ethics Statement

All experimental procedures were approved by the ethics committee of Shanghai Jiao Tong University and implemented according to the National Institutes of Health guidelines for the Care and Use of Laboratory Animals.

## Author Contributions

C-hJ designed research. C-rX, J-rL, S-wJ, LW, XZ, LX, X-mH, S-tL, H-jC, X-jF, and C-hJ performed research. S-tL, X-jF, and C-hJ contributed new reagents. LX and X-mH analyzed data. J-rL and S-wJ drew graphs. C-rX and C-hJ wrote the paper. All authors read and approved the manuscript.

## Funding

This study was sponsored by the Shanghai Pujiang Program (2019PJD031), Hospital Funded Clinical Research, Xinhua Hospital Affiliated to Shanghai Jiaotong University School of Medicine (21XHDB01), Natural Science Foundation of Zhejiang Province of China (LQ20H090015), Innovation Talent Plan of Zhejiang Provincial Department of Health (No. 2020RC012), and National Science Foundation of China (No. 81300994).

## Conflict of Interest

The authors declare that the research was conducted in the absence of any commercial or financial relationships that could be construed as a potential conflict of interest.

## Publisher’s Note

All claims expressed in this article are solely those of the authors and do not necessarily represent those of their affiliated organizations, or those of the publisher, the editors and the reviewers. Any product that may be evaluated in this article, or claim that may be made by its manufacturer, is not guaranteed or endorsed by the publisher.
